# Malformations of the tooth root in humans

**DOI:** 10.3389/fphys.2015.00307

**Published:** 2015-10-27

**Authors:** Hans U. Luder

**Affiliations:** Center of Dental Medicine, Institute of Oral Biology, University of ZurichZurich, Switzerland

**Keywords:** tooth root, abnormalities, humans, root dilaceration, taurodontism, odontodysplasia, dentin dysplasia type I, hypophosphatasia

## Abstract

The most common root malformations in humans arise from either developmental disorders of the root alone or disorders of radicular development as part of a general tooth dysplasia. The aim of this review is to relate the characteristics of these root malformations to potentially disrupted processes involved in radicular morphogenesis. Radicular morphogenesis proceeds under the control of Hertwig's epithelial root sheath (HERS) which determines the number, length, and shape of the root, induces the formation of radicular dentin, and participates in the development of root cementum. Formation of HERS at the transition from crown to root development appears to be very insensitive to adverse effects, with the result that rootless teeth are extremely rare. In contrast, shortened roots as a consequence of impaired or prematurely halted apical growth of HERS constitute the most prevalent radicular dysplasia which occurs due to trauma and unknown reasons as well as in association with dentin disorders. While odontoblast differentiation inevitably stops when growth of HERS is arrested, it seems to be unaffected even in cases of severe dentin dysplasias such as regional odontodysplasia and dentin dysplasia type I. As a result radicular dentin formation is at least initiated and progresses for a limited time. The only condition affecting cementogenesis is hypophosphatasia which disrupts the formation of acellular cementum through an inhibition of mineralization. A process particularly susceptible to adverse effects appears to be the formation of the furcation in multirooted teeth. Impairment or disruption of this process entails taurodontism, single-rooted posterior teeth, and misshapen furcations. Thus, even though many characteristics of human root malformations can be related to disorders of specific processes involved in radicular morphogenesis, precise inferences as to the pathogenesis of these dysplasias are hampered by the still limited knowledge on root formation.

## Introduction

Owing to the belief that periodontal regeneration is a recapitulation of the processes involved in root formation, these processes have recently gained increased attention from researchers (Luan et al., 2006; Huang and Chai, 2012; Xiong et al., 2013; Bosshardt et al., 2015). There is complete agreement that radicular development is controlled by Hertwig's epithelial root sheath (HERS) which is derived from the cervical loop of the enamel organ and determines root number, shape, and length. The end of crown morphogenesis comprising the cessation of enamel formation and the development of HERS is associated with the disappearance of the expression of mesenchymal fibroblast growth factor 10 (Fgf10) and of epithelial growth factor (Egf) receptor (Tummers and Thesleff, 2003; Yokohama-Tamaki et al., 2006; Fujiwara et al., 2009). Subsequently, HERS proliferates in an apical direction and induces the differentiation of odontoblasts and dentinogenesis. Other than during crown development, radicular dentinogenesis critically depends on nuclear factor Ic (Nfic) and transforming growth factor β (Tgfβ) signaling mediated by Smad4 (Huang and Chai, 2012; Xiong et al., 2013). On the first layer of root dentin, HERS cells deposit enamel matrix proteins (Xiong et al., 2013; Bosshardt et al., 2015). As far as the ultimate fate of HERS and its contribution to cementogenesis are concerned, opinions differ. There is general consensus that HERS disintegrates, thus forming the epithelial cell rests of Malassez and allowing mesenchymal cells of the dental follicle to gain access to the surface of the outermost dentin layer, where they differentiate into cementoblasts and form radicular cementum (Diekwisch, 2001; Luan et al., 2006; Huang et al., 2009; Huang and Chai, 2012; Xiong et al., 2013; Bosshardt et al., 2015). In addition, several researchers attribute to HERS a more active role in cementogenesis. There is evidence to suggest that some HERS cells undergo epithelial mesenchymal transition and differentiate into cementoblasts (Huang and Chai, 2012; Xiong et al., 2013; Bosshardt et al., 2015). HERS cells may even participate directly in cementogenesis and be embedded in the matrix of cellular cementum (Huang et al., 2009; Huang and Chai, 2012; Xiong et al., 2013). In the cervical root areas, cementoblasts incorporate a dense fringe of collagen fibers into the outer dentin and thus deposit the first layer of acellular cementum (Xiong et al., 2013; Bosshardt et al., 2015). Fringe fibers are subsequently elongated and mineralized under the control of tissue-nonspecific alkaline phosphatase (TNALP), while cementoblasts retreat from the advancing mineralization front (Bosshardt et al., 2015). In more apical root areas, cellular cementum comprising a mixture of predominantly intrinsic and scattered extrinsic collagen fibers (Shapey's fibers) is laid down. Other than during acellular cementum formation, cementoblasts, similar to osteoblasts, occasionally are embedded in the collagenous matrix as cementocytes (Xiong et al., 2013). Thus, the basic processes of root formation appear to comprise (1) the development of HERS associated with the transition from crown to root development, (2) apical growth of HERS associated with root elongation, (3) the induction of odontoblast differentiation and radicular dentinogenesis, (4) the disintegration of HERS and the initiation of cementogenesis as well as (5) formation of acellular and cellular cementum.

A special process confined to the development of multirooted teeth is the formation of the bi- or trifurcation. The critical structures for furcation formation seem to be tongue-shaped epithelial projections from the cervical loop of the enamel organ, which are already present but remain inactive during crown formation. Only when the root trunk is about to divide, these tongues proliferate and unite to form a continuous bridge. Similar to HERS in the periphery of the root, the epithelium of the bridges induces the differentiation of odontoblasts which subsequently produce the dentin at the floor of the pulp cavity, while bridge cells proliferate and grow apically in concert with the peripheral HERS (Schroeder, 1991). Even though epithelial bridges in the furcation area thus seem to behave similarly to HERS, it is not known whether bridge formation proceeds under the control of HERS. In this context a recent study of Kim et al. (2015) is notable. It indicated that other than in the crown, the induction of root odontoblast differentiation and particularly the formation of dentin in and subjacent to the furcation critically depends on osterix. Hence the developmental processes involved in furcation formation could well be subject to specific regulatory mechanisms.

Short and/or misshapen roots are most often due to hard tissue resorption. Such secondary abnormalities which usually affect single teeth or small groups of teeth are a frequent consequence of dento-periodontal traumas, local periodontal inflammation, or orthodontic tooth movement using excessive forces (Andreasen, 1985; Tronstad, 1988). Irrespective of the cause, root resorption constitutes an inflammatory reaction. Therefore, its consequences can hardly be considered a malformation and will not be dealt with further in this review. The most common true human root malformations can be subdivided into (1) disorders of root development alone and (2) disorders of root development associated with a general tooth dysplasia. Disorders of root development alone comprise:

- Premature arrest of root formation due to an extrinsic adverse effect
- Root dilaceration
- Root malformation associated with a cervical mineralized diaphragm/molar incisor malformation
- Short root anomaly
- Taurodontism

Disorders of root development associated with a general tooth dysplasia include:

- Double teeth
- Regional odontodysplasia
- Hypophosphatasia
- Dentin dysplasia type I

The same extrinsic adverse effects that entail root resorption can also lead to true developmental disorders, if they affect teeth during root morphogenesis. Most of these disorders are due to a premature arrest of radicular development as a consequence of a direct mechanical dento-periodontal trauma (Andreasen and Flores, 2007), local infection, radiation, or chemotherapy during the period of root morphogenesis (Jaffe et al., 1984; Sonis et al., 1990; Zarina and Nik-Hussein, 2005; Barbería et al., 2008). A local trauma can also indirectly affect developing permanent teeth when the insult primarily impacts on the primary predecessor. The effects of such indirectly acting traumas usually comprise enamel and (hidden) dentin hypoplasias in the crown, but in severe cases so-called root dilaceration, i.e., a serious root malformation, can ensue (Jafarzadeh and Abbott, 2007; Topouzelis et al., 2010). Root malformation associated with a cervical mineralized diaphragm (Witt et al., 2014), also designated as molar-incisor malformation (Lee et al., 2014, 2015), constitutes a recently described condition which is probably due to an extrinsic although so far unknown cause and affects all permanent first molars. Two additional malformations confined to the roots in only part of the dentition are the so-called short root anomaly (Lind, 1972) and taurodontism (Haskova et al., 2009; Dineshshankar et al., 2014). Short root anomaly is mainly observed in permanent maxillary central incisors, while taurodontism, i.e., a disorder of furcation formation, affects only multirooted teeth. In both conditions at least a genetic component has been presumed, but taurodontism also occurs as a sequel of radiotherapy, i.e., an extrinsic cause (Barbería et al., 2008). In mice primary disruption of the processes of root formation affecting all teeth has been observed as a consequence of various genetic defects, for example in the *Nfic* (Steele-Perkins et al., 2003; Park et al., 2007), *Ptc* (patched; Nakatomi et al., 2006), *Dkk1* (dickkopf-related protein 1; Han et al., 2011), *Osx* (osterix; Kim et al., 2015), *Smad4* (Huang and Chai, 2012), and *Wls* (wntless; Bae et al., 2015) genes. In humans, however, only two forms of clearly hereditary malformations of the roots alone seem to exist. The first one is associated with osteopetrosis due to genetic defects of *CLCN7* (encoding a chloride channel component; Xue et al., 2012), the second one is related to a defective *PLG* gene (encoding plaminogen; Tananuvat et al., 2014). In the absence of confirming evidence, both conditions thus far constitute isolated cases.

Among the disorders of root development associated with a general tooth malformation, those observed in cases of double teeth, i.e., geminated and fused teeth (Schuurs and van Loveren, 2000), as well as in regional odontodysplasia (Crawford and Aldred, 1989; Hamdan et al., 2004; Tervonen et al., 2004; Al-Tuwirqi et al., 2014) are due to unknown causes. In agreement with the designation, double teeth involve only two teeth, whereas regional odontodysplasia affects at least a group of contiguous teeth in a quadrant of the dentition. Generalized dysplastic roots occur as a result of hypophosphatasia (McKee et al., 2013) and in dentin dysplasia type I (O Carroll et al., 1991; Ansari and Reid, 1997; Toomarian et al., 2010), both of which are hereditary. The aim of the present review is to analyze the characteristic features of these root malformations in an attempt to derive which basic processes of root morphogenesis potentially are disrupted.

## Disorders of root development alone

### Premature arrest of root formation

Premature arrest of root formation most frequently results from a direct trauma to a developing tooth (Andreasen and Flores, 2007). If the traumatic insult irreversibly damages the apical periodontal ligament including HERS and the neurovascular supply, these tissues as well as the pulp become necrotic. This occurs particularly in cases of traumatic intrusion and less frequently due to lateral luxations and extrusions (Andreasen and Kahler, 2015). As a consequence root elongation and radicular dentinogenesis stop, leaving a shortened root with thin dentinal walls and a wide open apex. Such teeth are at increased risk of fractures and pose a challenge for the endodontic treatment. Therefore, efforts are made to close the apex using a so-called apexification (Shabahang, 2013) or even pulp revascularization (Wigler et al., 2013; Palit et al., 2014). In fact there is radiographic evidence indicating that root elongation and dentinogenesis can be resumed, forming a complete root with a closed apex (Kottoor and Velmurugan, 2013).

Treatment of childhood cancer using radio- or chemotherapy also entails developmental tooth alterations (Jaffe et al., 1984; Sonis et al., 1990; Minicucci et al., 2003; Zarina and Nik-Hussein, 2005; Barbería et al., 2008; Pedersen et al., 2012). Owing to their non-selective potential to destroy proliferating cells, the therapeutic agents affect developing teeth as well. Depending on the dosage of the therapy and the age of the patients, the resulting dental abnormalities range from agenesis of individual teeth over microdontia to shortened roots as a result of premature arrest of root formation. Interestingly, unlike short roots in cases of mechanical trauma, those due to early cancer therapy always exhibit closed apices. Thus, even if root elongation is halted prematurely, dentinogenesis seems to progress and to complete the root tip.

### Root dilaceration

**Figure 1:**
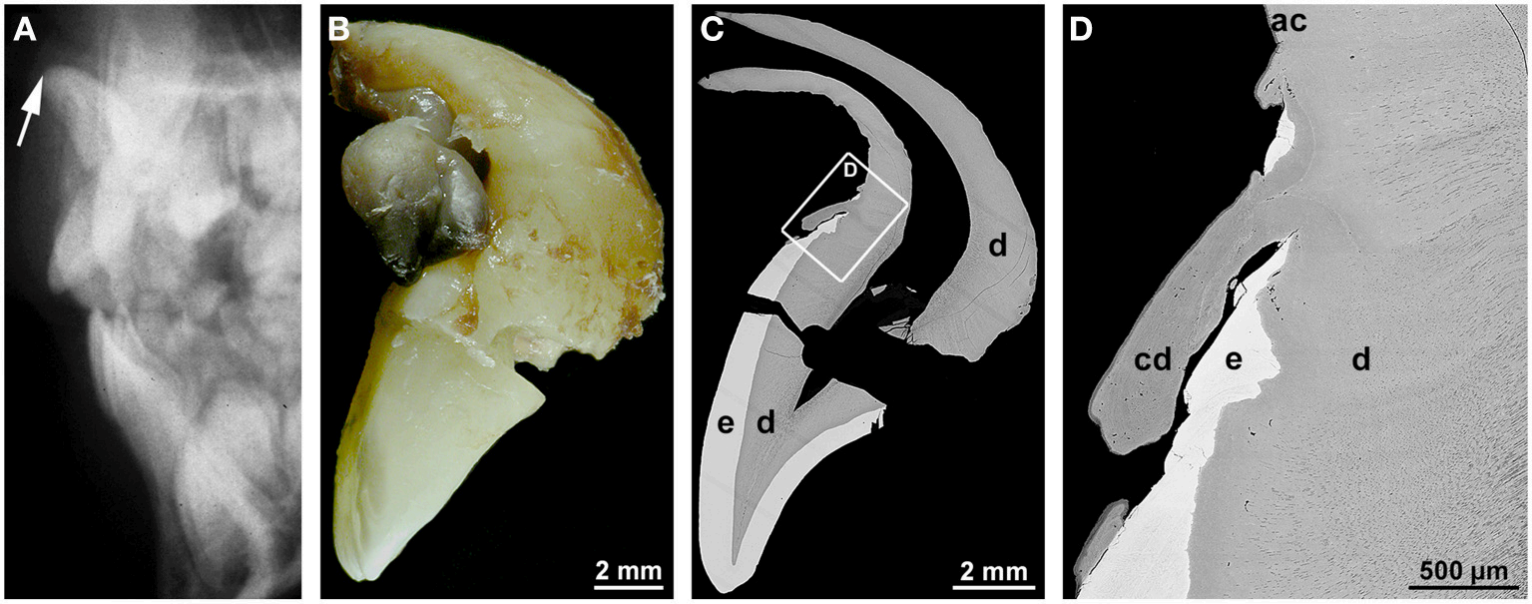
**Radiographic, macroscopic, and microscopic appearance of root dilaceration. (A)** A lateral cephalogram shows the position of the incisal edge of a permanent maxillary central incisor (arrow) approximately 3 years after an intrusive trauma to the primary predecessor at the age of about 4.5 years. **(B)** A mesial macroscopic view of the reassembled permanent incisor reveals a sharp bend of the tooth axis in the cervical region and a curved hook-shaped root. **(C,D)** Corresponding overview **(C)** and detail **(D)** backscattered electron micrographs from a labio-lingual ground section depict normal enamel (e) and dentin (d), a tongue of cellular dentin (cd) which probably resulted from a local disorganization of the enamel organ, and normal acellular cementum (ac). Original magnifications **(B)** 4x, **(C)** 45x, **(D)** 350x.

Dilaceration is most appropriately defined as a sharp bend of either the crown or root axis (Andreasen et al., 1971). This definition discriminates dilaceration from flexion which denotes a smooth physiologic or abnormal curvature of the root (Jafarzadeh and Abbott, 2007). Varying definitions of the condition might account for the wide range of percentages (0.42–98%) reported for the prevalence of dilaceration. Rather unexpectedly posterior teeth are more frequently affected than front teeth (Jafarzadeh and Abbott, 2007; Topouzelis et al., 2010). The cause of root dilaceration in molars and premolars is not entirely clear, although it is often seen in cases of eruption disorders when the forming roots of retained teeth encounter a cortical bone structure and subsequently are deflected (Marks and Cahill, 1984; Larson et al., 1994). In permanent front teeth dilaceration most often is a consequence of an indirect trauma to the primary predecessors (Jafarzadeh and Abbott, 2007; Topouzelis et al., 2010). The type of injury depends on the age of the patient. At early ages of 2–3 years the developing crown of the permanent tooth lies in a lingual position relative to the root of the primary predecessor. As a result, a luxation injury to the latter most likely hits the labial part of the permanent dental crown and causes enamel and (hidden) dentin hypoplasias. At the worst the formed part of the crown is dislocated to the lingual side, thus causing a crown dilaceration with a lingual angulation (Topouzelis et al., 2010). Root dilaceration occurs at later ages of 4–5 years, when the crown of the permanent successor is largely complete. At this stage of development, physiologic resorption of the primary tooth root has already started and the germ of the permanent tooth has moved to a position approximately in the axis of the predecessor. If the primary tooth at this stage is hit by an intrusive trauma, the crown as well as already formed radicular parts of the permanent tooth can be dislocated to the labial side (Topouzelis et al., 2010). As a consequence, a root dilaceration with a labial angulation ensues as is illustrated in Figure 1. In this example, the incisal edge of the affected central incisor pointed to the nasal floor (Figure 1A) and the formed root was hook-shaped (Figure 1B). As revealed by gaps in the enamel, the end of crown morphogenesis was temporarily disorganized, but coronal hard tissue formation apparently was resumed and completed (Figures 1C,D). Likewise the transition from crown to root development as well as the subsequent root elongation, dentinogenesis, and cementogenesis obviously progressed unaffected (Figures 1C,D). However, root morphogenesis appears to have followed the neuro-vascular supply which is derived from the infraorbital artery and nerve and runs down more or less vertically. As a consequence the flexed root developed (Topouzelis et al., 2010).

### Root malformation associated with a cervical mineralized diaphragm/molar incisor malformation

**Figure 2:**
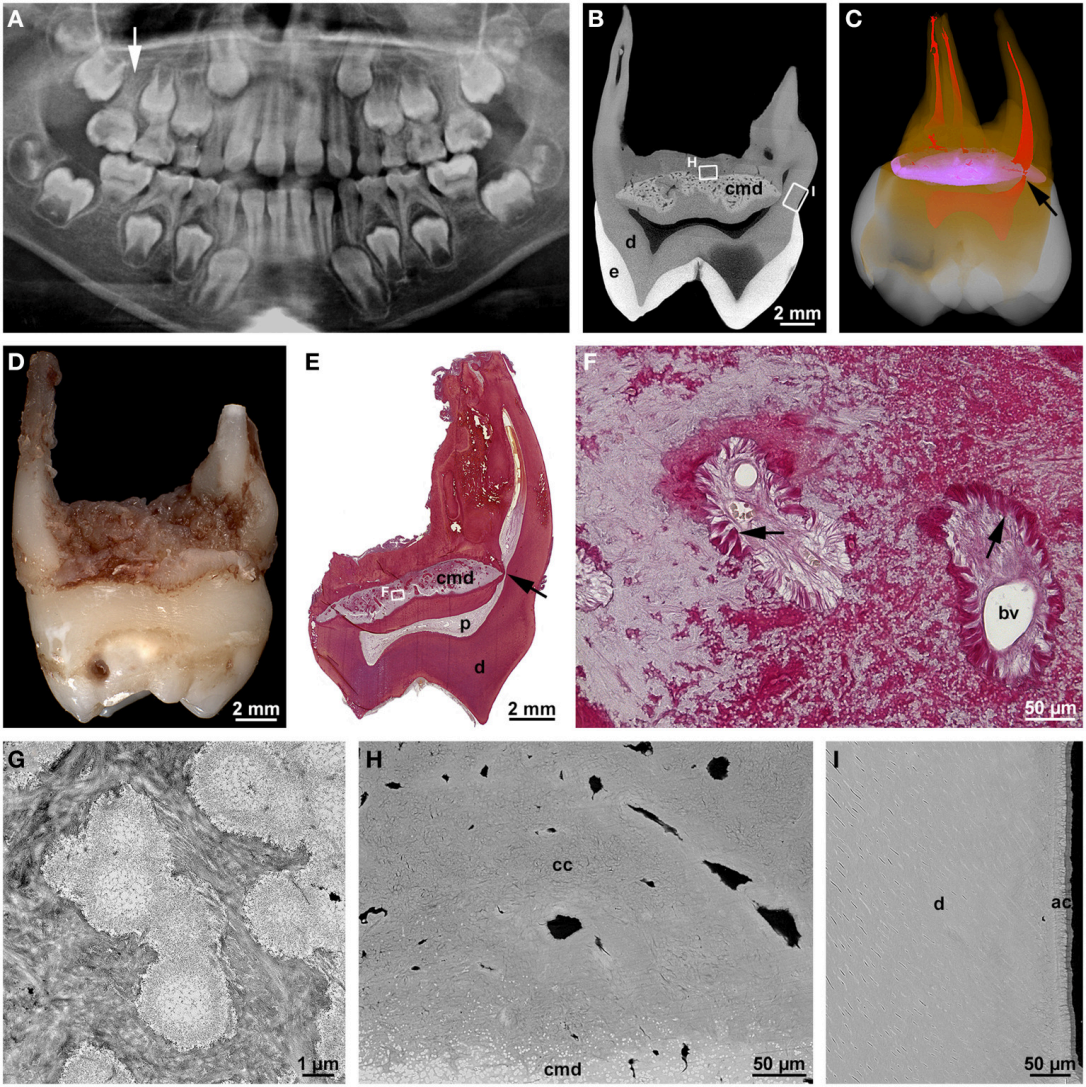
**Radiographic and microscopic characteristics of root malformation associated with a cervical mineralized diaphragm/molar incisor malformation**. **(A)** A panoramic radiograph taken from a boy at the age of 8 years 1 month reveals rudimentary roots of all four permanent first molars, of which the maxillary right one (arrow) served for the microCT and microscopic evaluation. **(B)** Bucco-lingual microCT section depicting normal enamel (e) and dentin (d) as well as the cervical mineralized diaphragm (cmd). **(C)** A buccal view of a three-dimensional microCT reconstruction shows enamel (white), dentin (orange), the CMD (violet), and the pulp (red); note the constricted lingual root canal (arrow) curving around the margin of the CMD. **(D)** Mesial macroscopic view of the extracted permanent maxillary right first molar. **(E)** An overview micrograph from a bucco-lingual section stained with resorcin-fuchsin reveals dentin (d), the pulp (p), and the lingual root canal (arrow) curving around the margin of the CMD. **(F)** A detail of the CMD from the same section shows two soft tissue canals containing blood vessels (bv) and connective tissue resembling periodontal ligament (arrows). **(G)** A transmission electron micrograph of the CMD depicts fine-granular, partly coalesced globules embedded in a collagenous matrix. **(H,I)** Backscattered electron micrographs from the roof of the furcation **(H)** and the outer surface of the lingual root **(I)** display the margin of the CMD, dentin (d) as well as cellular (cc) and acellular (ac) cementum. Original magnifications **(D)** 3.2x, **(E)** 4x, **(F)** 200x, **(G)** 13000x, **(H,I)** 1500x. **(A)** is reprinted from Witt et al. (2014) with permission from Elsevier.

At about the same time, an own study (Witt et al., 2014) and a report from South Korea (Lee et al., 2014) described a new type of root malformation which consistently affects the permanent first molars. Based on the observed distinguishing feature, we termed it root malformation associated with a cervical mineralized diaphragm (RM-CMD), while Lee et al. (2014) called it molar-incisor malformation (MIM), because these authors noted also involvement of primary second molars and permanent maxillary central incisors in some cases. Affected permanent molars exhibit inconspicuous crowns, short, tapered roots and slit-shaped pulp cavities of markedly reduced height (Figures 2A,D). The distinguishing microscopic feature is a roughly lens-shaped mineralized plate at the level of the cemento-enamel junction, which we called a cervical mineralized diaphragm (CMD; Figures 2B,C,E). It comprises densely calcified, sometimes coalesced globules embedded in a moderately mineralized collagenous matrix as well as a network of soft tissue canals containing large blood vessels and connective tissue resembling periodontal ligament (Figures 2F,G). On the basis of these microscopic features, we proposed that the CMD developed in response to an as yet unknown external insult and was derived from the dental follicle (Witt et al., 2014). In contrast, Lee et al. (2015) based on the immunohistochemical demonstration of dentin sialoprotein, collagen type XII, and osteocalcin concluded that the CMD originated mainly from the apical pulp, i.e., a derivative of the apical papilla, and partially from the dental follicle.

The course of the tubules in the cervical coronal dentin suggested that the CMD already existed when dentinogenesis approached the cemento-enamel junction. It seemed to constitute a mechanical obstacle interfering with the normal retraction of the odontoblasts (Witt et al., 2014). Similarly, it also affected the formation of the root canals which curved around the margins of the CMD and were markedly constricted (Figures 2B,C). Nevertheless, the outer parts of the root stumps were comprised of regular tubular dentin covered by a layer of acellular cementum (Figure 2I), whereas the roof of the furcation immediately subjacent to the CMD contained only cellular cementum and interspersed soft tissue canals (Figure 2H). Thus, the transition from crown to root development, the initial apical growth of HERS, and the induction of dentinogenesis and cementogenesis along the root periphery seem to have progressed more or less unaffected. However, in the furcation area dentinogenesis was completely disrupted and replaced by excessive formation of cellular cementum, although the epithelial tongues of HERS might have fused.

### Taurodontism

**Figure 3:**
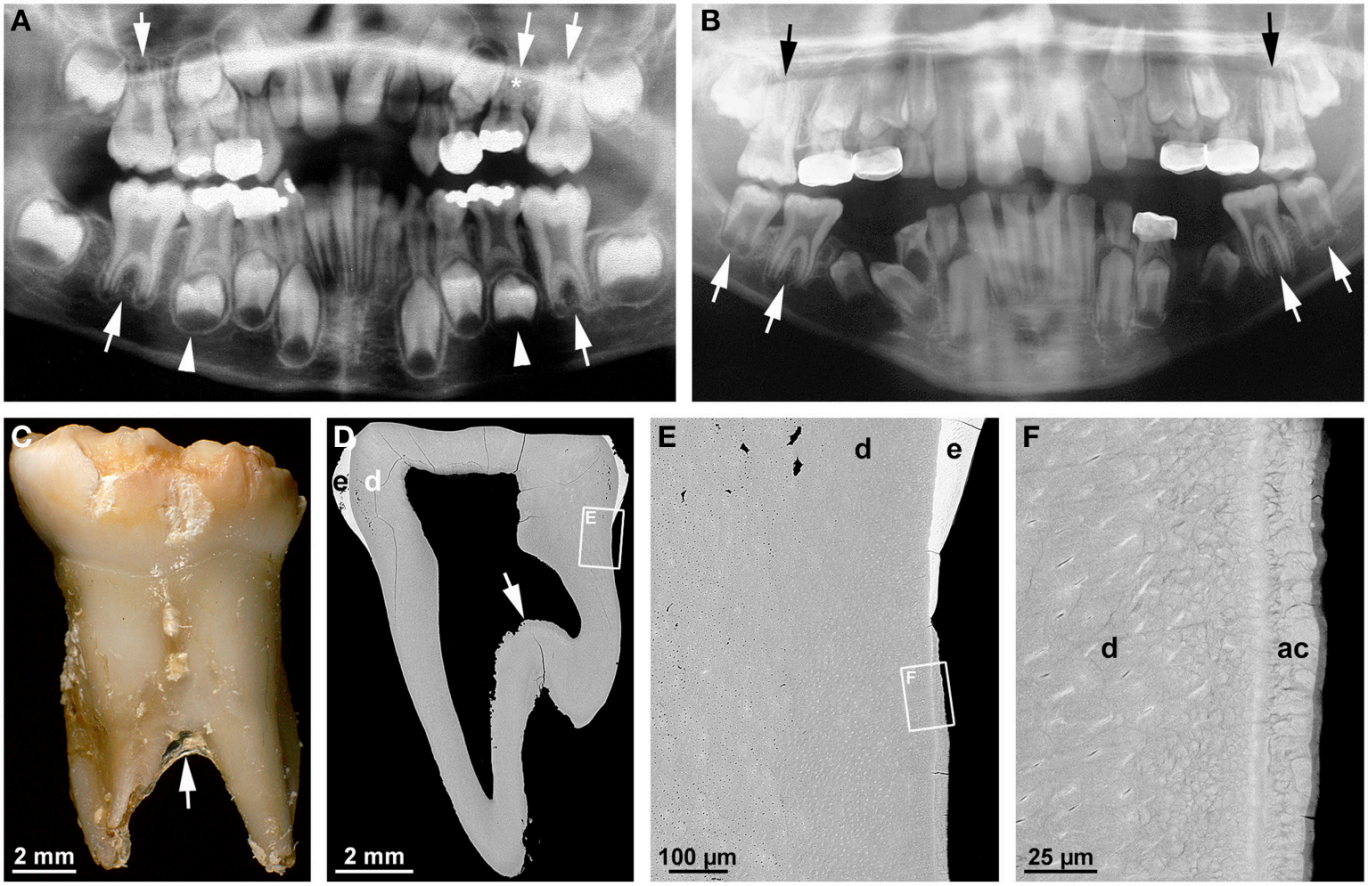
**Radiographic, macroscopic, and microscopic features of taurodontism. (A)** A panoramic radiograph from a case of isolated taurodontism reveals mesotaurodontism of all permanent first molars and primary maxillary second molars (arrows), hypertaurodontism of the primary mandibular second molars (arrow-heads), and agenesis of the permanent maxillary left second premolar (asterisk). **(B)** The panoramic radiograph from an 11 year-old boy affected by tricho-dento-osseous syndrome due to a mutation in the *DLX3* gene reveals hypotaurodontism of all permanent first and mandibular second molars (arrows). Note the low contrast between enamel and dentin as a consequence of amelogenesis imperfecta. **(C)** A buccal macroscopic view of the primary mandibular left second molar extracted from the same boy several years earlier shows the markedly apical location of the bifurcation (arrow) and the brittle, partially chipped enamel. **(D–F)** Overview **(D)** and detail **(E,F)** backscattered electron micrographs from a mesio-distal ground section of the primary molar depict the apical position of the floor of the pulp cavity (arrow), hypoplastic-hypomaturated enamel (e), normal dentin (d), and normal acellular cementum (ac). Original magnifications **(C)** 6x, **(D)** 50x, **(E)** 800x, **(F)** 4000x.

The term taurodontism denotes a feature of multirooted teeth characterized by apical displacement of the bi- or trifurcation (Figures 3A–C; Haskova et al., 2009; Dineshshankar et al., 2014). This is accompanied by a reduced or absent constriction at the cemento-enamel junction and an increased occluso-apical height of the pulp cavity (Figure 3D). Taurodontism is seen in both permanent and primary teeth, although less commonly in the latter (Figure 3A; Bafna et al., 2013). Its overall prevalence ranges from about 0.25 to 11.3% (Haskova et al., 2009). Depending on the severity it is classified as hypo- (mild), meso- (moderate), and hypertaurodontism (severe; Dineshshankar et al., 2014). Taurodontism arises when the formation of the epithelial bridges in the area of the future furcation is delayed (Haskova et al., 2009; Dineshshankar et al., 2014). The condition has been considered an atavistic trait, possibly because it was widespread in teeth of Neanderthals (Kupczik and Hublin, 2010). However, it also occurs as a consequence of childhood cancer treatment (Barbería et al., 2008). This is not particularly surprising when considering that both radiotherapy and chemotherapy are aimed at destroying proliferating tissues and, therefore, conceivably also impair growth of the epithelial tongues of HERS. Several associations between taurodontism and other dental and non-dental conditions reveal that a genetic component is involved in the etiology. Thus, taurodontism is a key feature of tricho-dento-osseous syndrome (TDO; ^1^OMIM#190320) which besides is characterized by kinky, curly hair during childhood and adolescence, hypoplastic-hypomaturation type amelogenesis imperfecta, and increased bone density due to dominant mutations of the homeobox gene *DLX3* (Figures 3B–F; Wright et al., 1997, 2008; Price et al., 1998). A *DLX3* mutation has also been reported to account for amelogenesis imperfecta hypoplastic-hypomaturation with taurodontism (AIHHT; OMIM#104510; Dong et al., 2005), although it has been disputed whether the described condition really constituted AIHHT or rather TDO with only minor hair and bone involvement (Price et al., 1999).

A microscopic examination of a primary mandibular second molar from a case of TDO demonstrates that apart from the apical location of the floor of the pulp cavity (Figure 3D), all components of the root including dentin and cementum are normal (Figures 3E,F). Thus, in humans *DLX3* does not seem to be involved in any process of root development except the formation of the furcation. This contrasts with the phenotype entailed by a neural crest deletion of *Dlx3* in mice, which revealed short molar roots and enlarged pulp chambers with thin dentinal walls but no obvious taurodontism (Duverger et al., 2012). Since the features associated with the *Dlx3* deletion were shown to be due to down-regulation of dentin sialophosphoprotein (Dspp), they appear to rather phenocopy the so-called shell teeth observed in dentinogenesis imperfecta type III (OMIM#125500) which is caused by mutations in the *DSPP* gene (MacDougall et al., 2006; Kim and Simmer, 2007). Concordance of the phenotypes in knock-out mice and humans, at least with respect to taurodontism, was observed as a result of defects of the *WNT10A* gene (Yang et al., 2015). Yang et al. (2015) speculated that loss-of-function mutations of *WNT10A* in Neanderthals could account for the widespread occurrence of taurodontism in these ancestors. Apart from taurodontism, genetic defects of *WNT10A* in modern humans cause also tooth agenesis and alterations in shape of the dental crowns but no other obvious tooth abnormalities. This combination of features suggests that *WNT10A* is important at the early initiation stage of odontogenesis as well as in the later stages of crown and root morphogenesis, but not in hard tissue formation (Yang et al., 2015).

### Short root anomaly

Short root anomaly (SRA) has first been described by Lind (1972) and recently reviewed by Valladares Neto et al. (2013). Its overall prevalence is about 0.6–2.4%, but it occurs about 2.5–3 times more often in females than males. By far the most frequently affected teeth are the permanent maxillary central incisors, while other teeth, mainly premolars, are more rarely involved. The precise etiology of SRA is unknown, although clear familial clustering suggests that a genetic component at least plays a role (Lind, 1972). Since the crowns of affected teeth are perfectly normal, the anomaly is detected incidentally on radiographs. It is characterized by plump, short (only little longer or even shorter than the crowns) roots of inconspicuous radiodensity, which lack any signs of antecedent hard tissue resorption. This suggests that the basic processes of root morphogenesis progress normally, but root growth in length, i.e., the apical growth of HERS, is deficient.

## Disorders of root development associated with a general tooth dysplasia

### Double teeth

**Figure 4:**
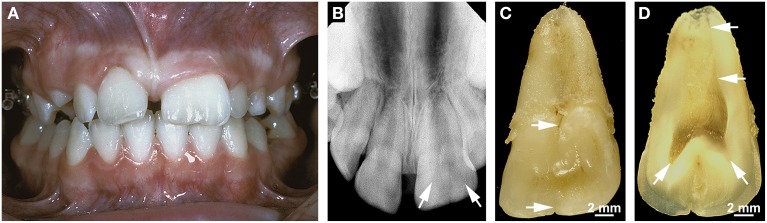
**Clinical, radiographic, and macroscopic appearance of gemination. (A)** An intraoral view of a geminated permanent maxillary left central incisor shows the size of the double tooth in comparison with that of the normal right central incisor. Note that the tooth count is correct if the oversized incisor is counted as one. **(B)** The apical radiograph from the same incisor reveals two faintly visible separate pulp horns (arrows). **(C)** A lingual macroscopic view of the double tooth shows a small notch in the incisal edge and a groove in the cervical tubercle (arrows) which continues as a shallow groove on the root. **(D)** A macroscopic view of the mesio-distally cut incisor demonstrates the partially divided coronal pulp and the single root canal (arrows). Original magnifications **(C,D)** 4x.

Double teeth result from the union of two adjacent teeth during odontogenesis. The etiology is unknown; presumed contributing factors are an evolutionary trend, trauma, environmental factors, and a hereditary component (Schuurs and van Loveren, 2000; Shashirekha and Jena, 2013; Hattab, 2014). Double teeth occur as two distinct entities which are referred to as gemination (with a prevalence of about 0.08–2.5%) and fusion (with a frequency of 0.1–0.85%; Shashirekha and Jena, 2013; Hattab, 2014). Gemination denotes a form of double teeth originating from the union of a regular and a supernumerary tooth, i.e., it reflects the incomplete splitting of one tooth germ. Fusion occurs when two regular tooth germs conjoin. Depending on the stage of development at which the union takes place, double teeth exhibit a broad crown with only an incisal/occlusal notch or labial/buccal groove and a single broad root (Figures 4A–C) or a partially divided crown and partially or completely separated roots (Schuurs and van Loveren, 2000; Hattab, 2014). Although, their validity has been doubted by Schuurs and van Loveren (2000), the classical criteria for discriminating gemination and fusion are a tooth count and the pulp anatomy of the conjoined teeth. When the oversized tooth is counted as one, the number of teeth is normal in cases of gemination and reduced in cases of fusion. Geminated teeth usually exhibit a partially or completely united pulp cavity (Figure 4D), while the pulp is completely divided in fused teeth. In cases of gemination the roots commonly are straight and broad and exhibit only a shallow groove in the area of the union (Figures 4B,C), whereas in cases of fusion the roots can be distorted because the germs of the conjoined teeth are not perfectly aligned (Schuurs and van Loveren, 2000). However, there are no indications that any component of the misshapen roots is defective. Thus, a normal HERS appears to develop from the cervical loop of the united enamel organs and to control all subsequent steps of radicular morphogenesis.

A condition related to but distinct from double teeth is an entity referred to as concrescence (Romito, 2004). Concrescence denotes a union of adjacent teeth by means of only radicular cementum. The etiology of such a union is unknown; trauma and a tight relationship of the neighboring tooth roots have been considered possible causative factors. Since concrescence can arise during or after root development (Romito, 2004), it is not always a true root malformation.

### Regional odontodysplasia

**Figure 5:**
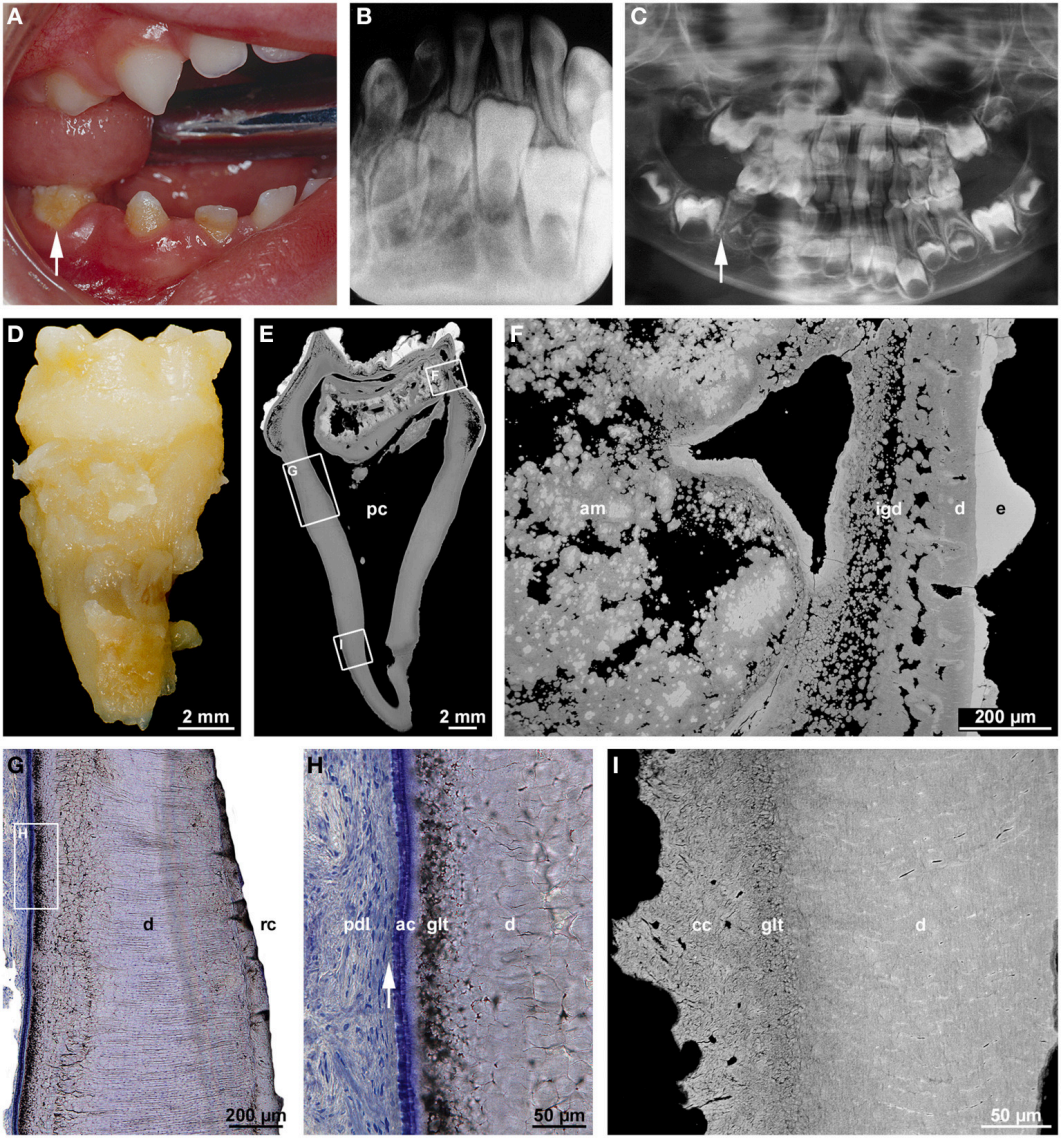
**Clinical, radiographic, and microscopic features of regional odontodysplasia**. **(A)** An intraoral view of the affected mandibular right quadrant taken from a boy at the age of 2 years 8 months shows the primary second molar (arrow) which was later examined microscopically. **(B)** Apical radiograph of the mandibular front at the age of 4 years 7 months illustrating the ghost-like appearance of the permanent incisors. **(C)** A panoramic radiograph at the age of 5 years reveals a single root in the primary mandibular right second molar (arrow) as compared to the normally spread roots in the contralateral tooth. **(D)** Macroscopic buccal view of the single-rooted primary mandibular second molar extracted at the age of 5 years 4 months. **(E,F)** An overview **(E)** and detail **(F)** backscattered electron micrograph of a bucco-lingual ground section show hypoplastic enamel (e), tubular dentin containing clefts (d), interglobular dentin (igd), and amorphous masses (am) consisting of densely calcified globules and cellular dentin. **(G,H)** An overview **(G)** and detail **(H)** light micrograph of the cerviacl root surface stained with toluidine blue depict the root canal (rc), normal dentin (d), the granular layer of Tomes (glt), acellular cementum (ac), and Sharpey's fibers (arrow) attaching the periodontal ligament (pdl) to the root. **(I)** Backscattered electron micrograph of the apical root region revealing normal dentin (d), the granular layer of Tomes (glt), and cellular cementum (cc) with signs of hard tissue resorption. Original magnification **(D,E)** 5x, **(F)** 75x, **(G)** 50x, **(H)** 200x, **(I)** 220x.

Regional odontodysplasia (RO) is so uncommon that it mostly has been characterized in reports of isolated cases (Gardner and Sapp, 1973, 1977; Gibbard et al., 1973; Sapp and Gardner, 1973; Kerebel and Kerebel, 1983; Fearne et al., 1986; Gerlach et al., 1998), although several reviews cover the literature from successive time periods (Crawford and Aldred, 1989; Hamdan et al., 2004; Tervonen et al., 2004; Al-Tuwirqi et al., 2014). RO is an apparently non-hereditary disorder of enamel and dentin mostly affecting all or part of a quadrant, sometimes also two or more quadrants, and more often the maxilla than the mandible. The etiology is unclear; local circulatory disorders, viral infections, or a neural disturbance have been considered the most likely causes. Patients usually seek medical help at an early age because of a delay or failure in tooth eruption or because of pain resulting from pulp infections. Affected teeth exhibit a rough, discolored crown surface (Figures 5A,D) and radiographically a characteristic “ghost-like” appearance (Figures 5B,C). Microscopically, dental enamel is hypoplastic and hypomineralized (Figures 5E,F). The hard tissue subjacent to the dentin-enamel junction often comprises thin layers of more or less regular tubular mantle dentin and interglobular dentin. Most of the space normally occupied by circumpulpal dentin and the pulp contains large voids as well as so-called amorphous masses of densely mineralized globules and less densely mineralized hard tissue which has been classified as cellular dentin (Figures 5E,F; Crawford and Aldred, 1989). No dentinal tubules exist in the dysplastic core of the crown, suggesting that the abnormal deposits do not constitute a mechanical obstacle for dentinogenesis. Rather odontoblasts must be assumed to have died for unknown reasons, after some circumpulpal dentin had been laid down.

The microscopic features probably account for the ghost-like radiographic appearance and the susceptibility to pulp infection in the absence of caries. Loss of pulp vitality might also be the reason why root formation often ends prematurely, leaving wide open apices (Gibbard et al., 1973; Fearne et al., 1986; Crawford and Aldred, 1989; Hamdan et al., 2004). However, if the pulp remains vital long enough, radicular morphogenesis although somewhat delayed can be completed and result in a closed apex (Gardner and Sapp, 1973; Gibbard et al., 1973; Gerlach et al., 1998; Spini et al., 2007). This was also true for the primary mandibular right second molar of the case shown in Figure 5. In agreement with previous reports (Crawford and Aldred, 1989; Gerlach et al., 1998; Hamdan et al., 2004; Carlos et al., 2008), radicular dentin was much closer to normal than coronal dentin and in particular did not contain amorphous masses (Figures 5E,G–I). In the periphery, a granular layer of Tomes could be identified, and dentinal tubules traversed the entire wall of the root canal which, however, was abnormally wide and contained some scattered denticles. As in the crown, dentinogenesis obviously ended before the normal thickness of radicular dentin was attained. Whether, this occurred because the pulp died due to an infection or was caused by something else remains obscure. Irrespective of the premature termination of dentinogenesis, both accellular (Figures 5G,H) and cellular (Figure 5I) cementum were normal in appearance. This agrees with earlier reports (Gardner and Sapp, 1973; Gibbard et al., 1973) although there are also studies indicating that cementum is thin (Carlos et al., 2008) or even absent in places (Crawford and Aldred, 1989). Thus, even if possibly delayed, the transition from crown to root morphogenesis, apical growth of HERS and root elongation as well as the induction of radicular dentinogenesis and cementogenesis seem to progress properly in RO. Strikingly, however, a bifurcation dividing the root trunk into normally spread mesial and distal roots failed to form in the presented primary mandibular second molar (Figures 5C,D). Obviously, the epithelial projections of HERS did not unite and a furcation failed to form. This can also occur in the absence of other dental abnormalities although single-rooted primary molars are rare (Haridoss et al., 2014).

### Hypophosphatasia

Hypophosphatasia (HPP) is caused by homozygous, compound heterozygous, or heterozygous loss-of-function mutations in the *ALPL* gene encoding tissue-nonspecific alkaline phosphatase (TNALP). This enzyme controls biomineralization of organic matrices in bones and teeth by cleaving inorganic pyrophosphate which acts as a strong inhibitor of mineral crystal deposition (McKee et al., 2011, 2013). Based on the age at onset, HPP is classified into a perinatal, infantile (OMIM#241500), childhood (OMIM#241510), and adult (OMIM#146300) form. Clinical features include mineralization disorders of bones and teeth, which manifest themselves as rickets and early loss of teeth. These manifestations vary tremendously from severe (lethal) in the perinatal/infantile form to mild in the adult form of the disease. An additional form lacking any signs of skeletal involvement and showing only the dental features is referred to as odontohypophosphatasia.

**Figure 6:**
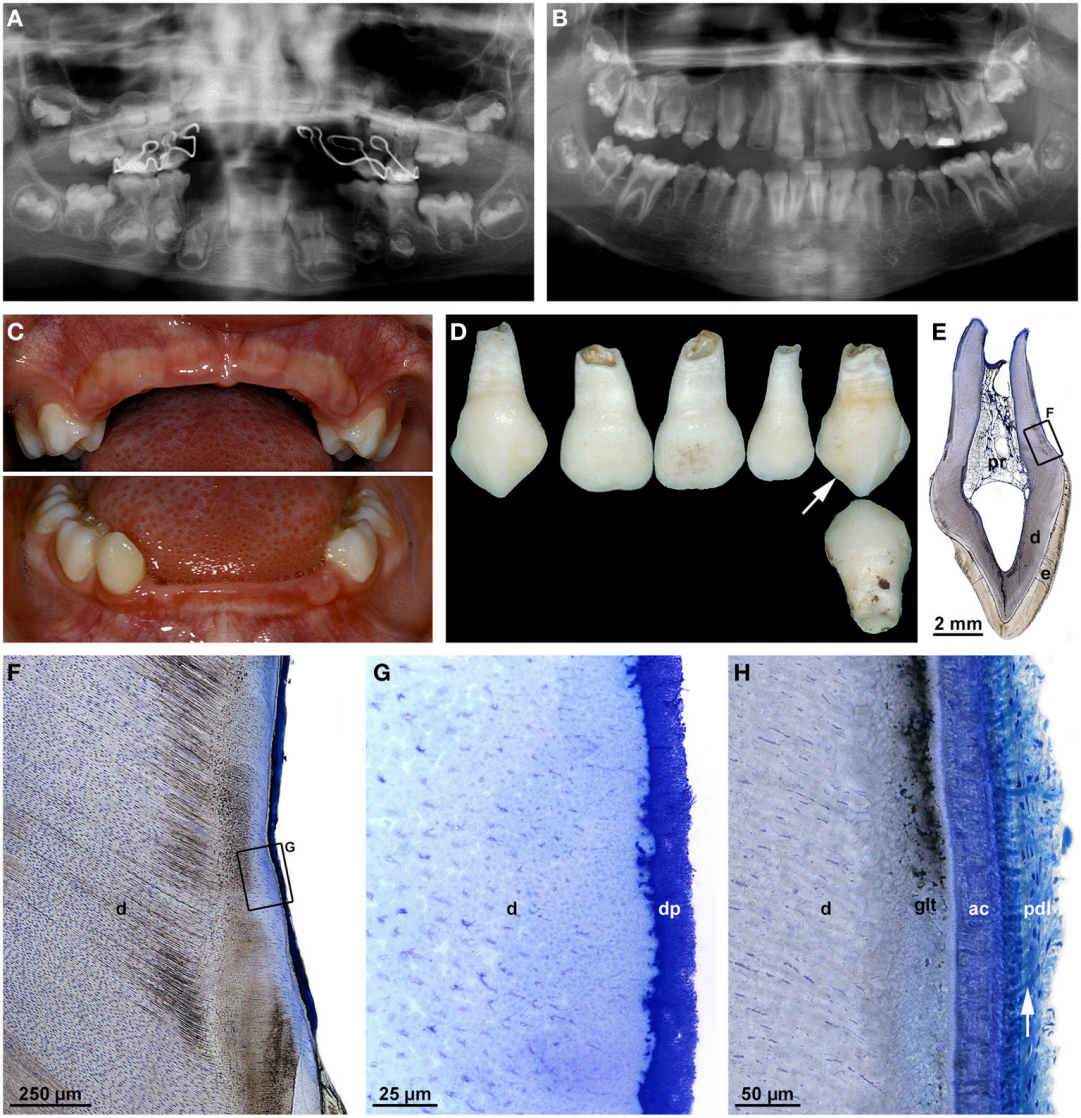
**Clinical, radiographic, and microscopic features of hypophosphatasia. (A,B)** Panoramic radiographs taken from a boy at the ages of 5 years 6 months **(A)** and 10 years 8 months **(B)**; the early radiograph **(A)** shows the remaining primary molars as well as the germs of the permanent teeth, metal-dense structures in the maxilla originate from clasps of a prosthesis. The later radiograph **(B)** reveals aberrant shapes of the crowns and roots of the permanent teeth. **(C)** Intraoral view at the age of 3 years following the premature exfoliation of 11 of 12 primary front teeth. **(D)** Recovered exfoliated primary teeth, of which the maxillary left canine (arrow) was used for the microscopic investigation. **(E–G)** Overview **(E)** and detail **(F,G)** light micrographs from a bucco-lingual ground section stained with toluidine blue show normal enamel (e) and dentin (d), remnants of the pulp (pr), and dental plaque (dp). **(H)** A comparable detail micrograph from the root surface of a healthy primary canine reveals normal dentin (d), the granular layer of Tomes (glt), acellular cementum (ac), and Sharpey's fibers (arrow) attaching the periodontal ligament (pdl) to the root. Original magnifications **(E)** 5x, **(F)** 50x, **(G)** 400x, **(H)** 200x.

The case illustrated in Figure 6 is an example of odontohypophosphatasia which exhibits most of the typical dental features described in previous case reports (Baer et al., 1964; El-Labban et al., 1991; Lundgren et al., 1991; Chapple, 1993; Olsson et al., 1996; Lepe et al., 1997; Hu et al., 2000; Van den Bos et al., 2005). At the age of 3 years the boy was referred to a pediatric dentist because several primary front teeth had spontaneously exfoliated, before their roots were fully formed (Figures 6C,D). No radiographic signs of rickets could be detected. Only after a histological examination of the exfoliated primary teeth had revealed complete absence of acellular cementum (Figures 6F,G), laboratory tests were made which revealed markedly lowered serum concentrations of TNALP (32 U/l) and markedly elevated concentrations of pyridoxal-5-phosphate (317.1 μg/l). Also urinary levels of phosphoethanolamine, i.e., a hallmark of HPP (Lundgren et al., 1991), were increased. Complete aplasia of cementum has also been observed in earlier investigations (Hu et al., 2000), but a majority of authors described cementum as thin and only partially missing (Baer et al., 1964; El-Labban et al., 1991; Lundgren et al., 1991; Olsson et al., 1996). Notably, cellular cementum seems to be less severely affected than acellular cementum (McKee et al., 2013), possibly because formation of the cellular variety is rather insensitive to alterations in pyrophosphate (Zweifler et al., 2015). Instead of cementum, a thick subgingival dental plaque covered the root surface of the primary teeth in the presented patient (Figure 6G). Bacterial plaque on the roots of affected teeth has also been observed previously and was even considered responsible for the lack of periodontal support (El-Labban et al., 1991), but this inference was disputed by Olsson et al. (1996). Likewise in agreement with previous reports (Baer et al., 1964; Lundgren et al., 1991; Hu et al., 2000; Van den Bos et al., 2005), the radicular dentin of the exfoliated teeth was regularly tubular (Figures 6F,G) and the size of the pulp chamber corresponded fairly well to the stage of dental development (Figures 6A,D,E). Thus, it would appear that cementogenesis is particularly sensitive to a deficiency in TNALP, while dentinogenesis and root growth are less vulnerable (McKee et al., 2011). However, as suggested by a radiograph taken from the presented boy at the age of 10 years 8 months (Figure 6B), this may vary between primary and permanent teeth. While no permanent teeth had been lost by then and, hence, their periodontal support was obviously sufficient, both crowns and roots of the canines, premolars, and molars were malformed. This raises the question, whether TNALP in addition to mineralization of dental hard tissues directly or indirectly also affects tooth morphogenesis.

### Dentin dysplasia type I

**Figure 7:**
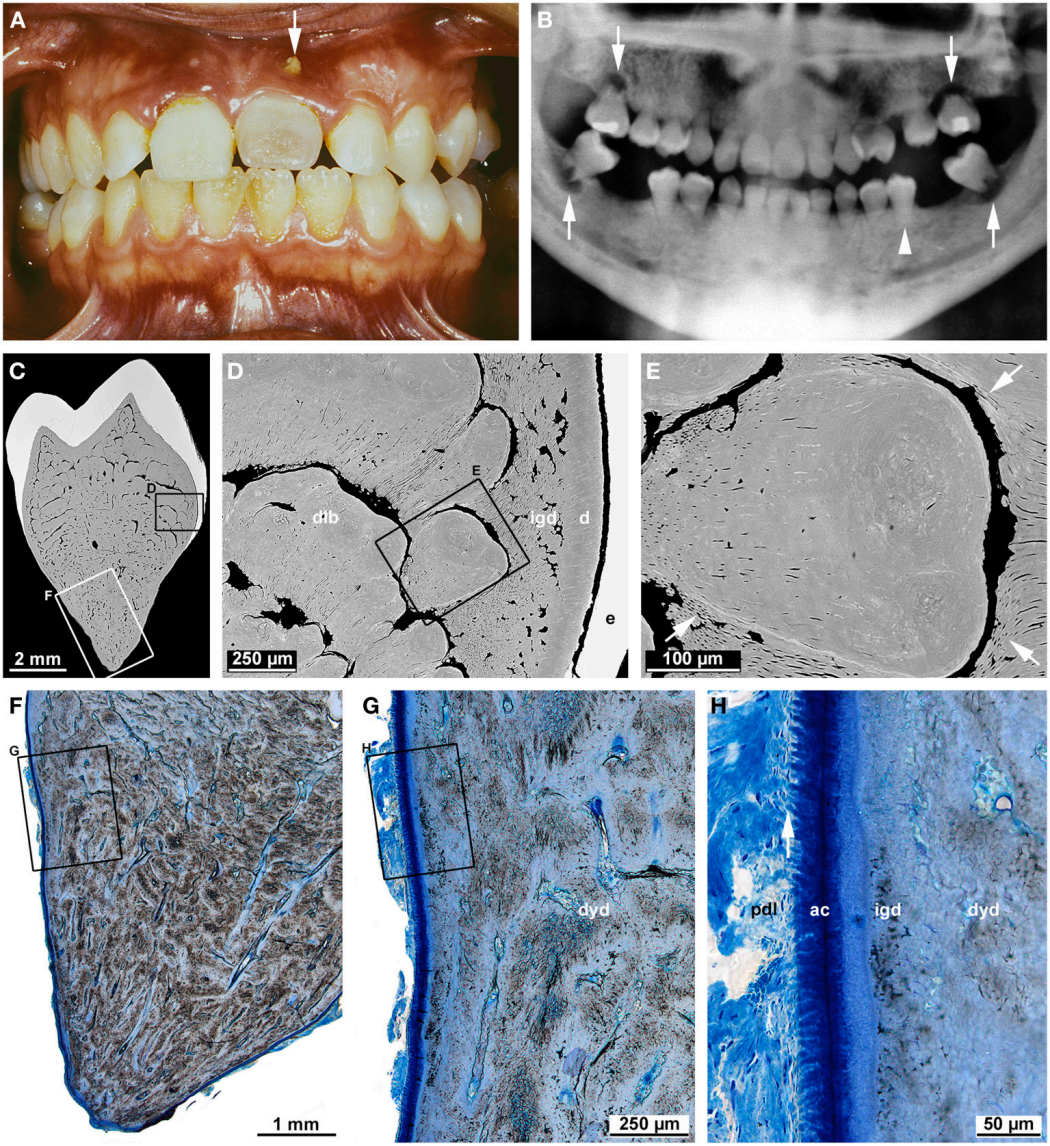
**Clinical, radiographic, and microscopic characteristics of dentin dysplasia type I**. **(A)** An intraoral view of a female patient at the age of 16 years 3 months shows a slightly opalescent permanent maxillary central incisor with a fistula (arrow). **(B)** A panoramic radiograph taken at the same age reveals short roots in all permanent teeth as well as only rudimentary furcations and apical radiolucencies in the second molars (arrows); the mandibular left second premolar (arrow-head) served for the microscopic investigation. **(C–E)** Overview **(C)** and detail **(D,E)** backscattered electron micrographs from a bucco-lingual ground section of the premolar depict normal enamel (e) and dentin (d), interglobular dentin (igd), and denticle-like bodies (dlb). In the detail **(E)**, note the dentinal tubules (arrows) arising from the periphery and curving around the denticle-like body. **(F–H)** An overview **(F)** and details **(G,H)** of the root surface from the same ground section stained with toluidine blue reveal dysplastic dentin (dyd), interglobular dentin (igd), acellular cementum (ac), and Sharpey's fibers (arrow) attaching the periodontal ligament (pdl) to the root. Original magnifications **(C)** 10x, **(D)** 100x, **(E)** 350x, **(F)** 12.5x, **(G)** 50x, **(H)** 200x.

Dentin dysplasia type I (DDI; OMIM#125400) is a heritable dentin disorder transmitted as an autosomal dominant trait (O Carroll and Duncan, 1994). However, the causative genetic defect has not been elucidated so far. The frequency of DDI is about 1 in 100,000 (Kim and Simmer, 2007). For this reason, knowledge on this disorder mainly originates from reports of isolated cases (Wesley et al., 1976; Kalk et al., 1998; Vieira et al., 1998; Neumann et al., 1999; Shankly et al., 1999; Özer et al., 2004; Da Rós Gonçalves et al., 2008; Rocha et al., 2011), although a few reviews (O Carroll et al., 1991; Ansari and Reid, 1997; Toomarian et al., 2010) are also available. The case shown in Figure 7 illustrates the typical findings. Clinically, the crowns of affected teeth are usually normal in shape and color, but sometimes also slightly opalescent (Figure 7A). Radiographs reveal largely or completely obliterated pulp chambers and short, often pointed roots with apical radiolucencies in the absence of caries (Figure 7B). Based on these radiographic features, O Carroll et al. (1991) proposed a subdivision into four forms. However, this classification does not allow an unambiguous assignment of the presented case. Microscopic examination shows that in the crown, the enamel and a thin layer of dentin subjacent to the dentin-enamel junction is completely normal. Further inside, hard tissue comprises some still tubular interglobular dentin, but the bulk of the space normally occupied by the innermost circumpulpal dentin and pulp cavity is filled with roundish calcified bodies partially separated by crescent-shaped soft tissue spaces (Figures 7C–E). These calcified bodies are variably referred to as whorls (Wesley et al., 1976; O Carroll and Duncan, 1994) or denticles (Ranta et al., 1993). Shields et al. (1973) even considered them true denticles, which entailed the conclusion that their formation was induced by displaced fragments of a disintegrated HERS (Ranta et al., 1993). However, a close look at the denticle-like structures (Figure 7E) reveals that they lack the typical features of true denticles such as an epithelial core and tubules. Dentinal tubules rather appear to arise from the peripheral normal dentin and to curve around the calcified bodies. This suggests that normal odontoblasts attempt to lay down dentin, but on their way back from the advancing formation front are blocked and shunted by the preexisting ectopic obstacles in the dental papilla. Nevertheless, the cause of the ectopic hard tissue formation in the dental papilla remains obscure.

In the root, individual denticle-like structures cannot be identified. Rather a mass of dysplastic hard tissue and interspersed small soft tissue canals fill the entire core of the root. In the periphery, however, thin layers of regular and interglobular dentin as well as acellular cementum with inserting periodontal Sharpey's fibers are still present (Figure 7H). Thus, it seems that the transition from crown to root formation is unaffected and the apical growth of HERS as well as the induction of radicular dentinogenesis and cementogenesis start normally, but are prematurely halted by the ectopic hard tissue formed in the dental papilla. In the permanent second molars of the presented case, this apparently occurred shortly after a futile attempt to build a furcation (Figure 7B).

### Dentinogenesis imperfecta types I, II, and III, X-linked hypophosphatemia

Except for RO and DDI, dentin disorders do not appear to entail radicular malformations characterized by deviations in root length or shape, but rather alterations in pulp cavity dimensions. Dentinogenesis imperfecta type I associated with osteogenesis imperfecta (OMIM#166200) is caused by genetic defects of *COL1A1* and *COL1A2*, i.e., the two genes encoding the α1 and α2 chains of type I collagen. Dentinogenesis imperfecta type II (OMIM#125490) results from mutations in the *DSPP* gene which encodes dentin sialophosphoprotein, i.e., a non-collagenous component of the dentin matrix. Both forms of dentinogenesis imperfecta are characterized by early complete obliteration of the pulp cavity including the root canals. In contrast so-called shell teeth affected by dentinogenesis imperfecta type III (OMIM#125500) which is also caused by genetic defects of *DSPP*, exhibit excessively large pulp cavities (MacDougall et al., 2006; Kim and Simmer, 2007). X-linked hypophosphatemia (OMIM#307800) is due to mutations in the *PHEX* (phosphate-regulating gene with homologies to endopeptidases on the X chromosome) gene. Affected teeth exhibit hypomineralized dentin characterized by large amounts of so-called interglobular dentin (partly confluent globules of mineralized dentin separated by interstices of unmineralized matrix) and enlarged pulp cavities. These are sometimes mistaken as taurodontism although the furcation is not displaced apically (McKee et al., 2013).

## Discussion

A summarizing comparison of the described most common human root dysplasias and the basic processes of root formation (Table 1) shows that the development of HERS at the transition from crown to root morphogenesis constitutes a particularly robust process, whereas apical growth of HERS associated with root elongation as well as the formation of the furcation in multirooted teeth seem to be rather susceptible to various intrinsic and extrinsic adverse effects.

**Table 1:** **Summary of the most common human root malformations as against potentially affected processes of root development**.

**Root malformation**	**Processes of root formation**						
	**Hertwig's epithelial root sheath**				**Dentinogenesis**	**Formation of furcation**	
	**Formation (crown-root transition)**	**Apical proliferation (root elongation)**	**Induction of odontoblast differentiation**	**Disintegration, cementogenesis**		**Epithelial bridge formation**	**Interradicular dentinogenesis, cementogenesis**
Short roots due to							
direct trauma	Unaffected	Disrupted	Disrupted	Continues	Disrupted	n.a.^a^	n.a.^a^
radiotherapy	Unaffected	Disrupted	Disrupted	Continues	Continues	Delayed	Unaffected (?)
chemotherapy	Unaffected	Disrupted	Disrupted	Continues	Continues	Delayed (?)	Unaffected (?)
Root dilaceration	Delayed (?)	In wrong direction	Unaffected	Unaffected	Unaffected	Unaffected (?)	Unaffected (?)
Taurodontism	Unaffected	Unaffected	Unaffected	Unaffected	Unaffected	Delayed	Unaffected
Short root anomaly	Unaffected	Impaired	Unaffected	Unaffected	Unaffected	n.a.^a^	n.a.^a^
Root malformation with CMD	Unaffected	Impaired	Unaffected	Unaffected	Mechanically impaired	Disturbed	d^b^: disrupted c^c^: excessive
Double teeth	Unaffected	Unaffected	Unaffected	Unaffected	Unaffected	n.a.^a^	n.a.^a^
Regional odontodysplasia	Unaffected	Impaired	Unaffected (?)	Unaffected	Impaired	Impaired	Impaired (?)
Hypophosphatasia	Unaffected	Unaffected	Unaffected	Dis^d^: unaffected (?) ac^e^: impaired cc^f^: unaffected (?)	Impaired (?)	Unaffected	Impaired (?)
Dentin dysplasia type I	Unaffected	Impaired	Unaffected	Unaffected	Mechanically Impaired	Impaired (?)	d^b^: mechanically impaired

a *not applicable, because malformation largely occurs in single rooted teeth*;

b *d, dentinogenesis*;

c *c, cementogenesis*;

d *dis, disintegration*;

e *ac, acellular cementum formation*;

f cc, cellular cementum formation.

The transition from crown to root formation and the concomitant development of HERS do not appear to be disrupted in any of the described radicular dysplasias. Thus, truly rootless teeth seem to be extremely rare in humans. Among the impacts affecting the apical proliferation of HERS, direct mechanical traumas to erupted immature teeth occur too late to exert influence on crown-root transition. On the other hand as revealed by the cases of root dilaceration, the effect of earlier indirect mechanical traumas via the primary predecessor do not appear to be harsh enough to completely disrupt HERS development, probably because the germ of the permanent tooth is rather well protected by the cushion of follicular soft tissues. Conceivably, early childhood cancer treatment using radiotherapy or chemotherapy could affect crown-root transition, but to the best of my knowledge no examples seem to exist in the literature. Considering that defects of the *Nfic* gene in mice completely prevent root formation (Steele-Perkins et al., 2003), human hereditary abnormalities associated with a radicular dysplasia are candidates for an effect on crown-root transition. In fact O Carroll et al. (1991) and O Carroll and Duncan (1994) displayed a graphical sketch of a tooth affected by DDI which virtually lacks roots. However, radiographs from real cases reveal at least small root stumps, suggesting that the designation “rootless teeth” in cases of DDI is not quite appropriate.

Apical growth of HERS and the associated root elongation turn out to be one of the most susceptible processes of radicular morphogenesis (Table 1). Hence shortened roots would be the most prevalent malformation in humans, which agrees with clinical experience. However, the undisputable success of attempts at revascularization of necrotic teeth after dental injuries (Kottoor and Velmurugan, 2013) suggests that even the premature arrest of root growth is not necessarily as irreversible as previously thought. Intimately related to the apical proliferation of HERS are the induction of odontoblast differentiation and the subsequent radicular dentinogenesis. Therefore, the development of new odontoblasts from the ectomesenchymal cells of the dental papilla inevitably comes to a halt when root elongation is disrupted. However, further dentinogenesis also depends on the vitality of the pulp. If this is not challenged as in the case of radiotherapy or chemotherapy, dentinogenesis can continue even if root growth is arrested. Conversely, in cases of radicular dysplasias associated with enlarged root canals such as RO, it is often unclear whether the enlargement of the pulp cavity is due to impairment of dentinogenesis or the devitalization of the pulp, for example as a result of an infection. A remarkable observation in cases of severe dentinal disorders such as RO and DDI was that at least a thin peripheral layer of coronal and radicular dentin was normal. This suggests that the differentiation of odontoblasts is unaffected and dentinogenesis starts normally, until it encounters a mechanical obstacle (as in DDI) or is arrested for some unknown reason (as in RO).

Among the described human root malformations, HPP is the only one affecting cementogenesis, in particular the development of acellular cementum (Table 1). HPP is caused by mutations in the *ALPL* gene encoding tissue-nonspecific alkaline phosphatase (TNALPL) and the effect on cementogenesis seems to be due to defective mineralization resulting from a disrupted regulation of extracellular pyrophosphate cleavage (McKee et al., 2013). Although, a deficiency in TNALP can also entail consequences in dentin, the impact on cementum formation seems to be independent of that on dentinogenesis, as hypoplasia or even aplasia of cementum can occur in combination with completely normal radicular dentin. However, it remains mysterious how a disturbance of mineralization due to excessive concentrations of inorganic pyrophosphate can lead to complete absence of acellular cementum.

The processes involved in formation of the bi- or trifurcation in multirooted teeth appear to be particularly susceptible to extrinsic as well as genetic and other intrinsic influences (Table 1). In most conditions the formation of the epithelial bridges in the area of the future furcation is delayed, probably because epithelial proliferation is impaired. However, once the bridges are completed, the subsequent interradicular dentinogenesis and cementogenesis apparently can progress unaffected. Such a combination of a delay in furcation formation and normally progressing other processes of root development results in taurodontism which occurs as an isolated trait (Haskova et al., 2009), as part of hereditary syndromes (Wright et al., 1997; Yang et al., 2015), or as a feature accompanying consequences of radiotherapy and chemotherapy (Barbería et al., 2008). In RM-CMD/MIM the situation is exactly reversed: The roots of affected permanent first molars separate in an abnormally coronal position. Based on the microscopic structure of the misshapen furcation it is impossible to unambiguously derive whether epithelial bridges ever develop. Irrespectively the induction of odontoblast differentiation and dentinogenesis at the roof of the furcation and in the interradicular area are completely suppressed and no regular root trunk is formed. The most drastic disruption of furcation development leading to single-rooted posterior teeth apparently can occur in RO and most likely also in DDI. Whereas in RO the cause of the absent furcation formation is obscure, the masses of dysplastic hard tissue occupying the entire core of teeth affected by DDI conceivably interfere with the development of the epithelial bridges.

Thus, while many characteristics of the described most common human root dysplasias can be assigned to defects in specific processes of root development, the many question marks contained in Table 1 indicate that inferences as to the precise pathogenesis of root malformations are rather speculative, because the processes involved in radicular morphogenesis and particularly in furcation development are incompletely understood.

## Author's contribution to figures and ethical framework

All radiographs except the one shown in Figure 2A (Figures 1A, 3A,B, 4B, 5B,C, 6A,B, 7B) and intraoral photographs (Figures 4A, 5A, 6C, 7A) have been taken by dental technicians at the Center of Dental Medicine, University of Zurich. The radiograph shown in Figure 2A has been supplied by a private dental practice in Germany. All macroscopic photographs (Figures 1B, 2D, 3C, 4C,D, 5D, 6D), microCT reconstructions (Figures 2B,C), and micrographs (Figures 1C,D, 2E–I, 3D–F, 5E–I, 6E–H, 7C–H) have been made by the author.

Written informed consent regarding the usage of the clinical documentation and processing of extracted teeth for teaching and scientific research purposes was obtained from the patients or their parents upon admission to the Center of Dental Medicine, University of Zurich. This procedure was approved by the institutional ethics committee (Ethics Committee of the Canton of Zurich, Switzerland) provided that no identification of patients was possible. Therefore, all case evaluations were performed in an anonymized way.

### Conflict of interest statement

The Reviewer, Dr Claudio Cantù, declares that, despite being affiliated to the same institution as the author, Dr Hans U. Luder, the review process was handled objectively. The author declares that the research was conducted in the absence of any commercial or financial relationships that could be construed as a potential conflict of interest.

## Footnotes

^1^OMIM: Online Mendelian Inheritance in Man®; http://omim.org/

## Abbreviations

DDI: dentin dysplasia type I
HERS: Hertwig's epithelial root sheath
HPP: hypophosphatasia
MIM: molar incisor malformation
RM-CMD: Root malformation associated with a cervical mineralized diaphragm
RO: regional odontodysplasia
SRA: short root anomaly
TNALP: tissue-nonspecific alkaline phosphatase.

## References

[B1] Al-Tuwirqi A. Lambie D. Seow W. K. (2014). Regional odontodysplasia: literature review and report of an unusual case located in the mandible. Pediatr. Dent. 36, 62–67. 24717712

[B2] Andreasen F. M. Kahler B. (2015). Pulpal response after acute dental injury in the permanent dentition: clinical implications - a review. J. Endod. 41, 299–308. 10.1016/j.joen.2014.11.015 25601716

[B3] Andreasen J. O. (1985). External root resorption: its implication in dental traumatology, paedodontics, periodontics, orthodontics and endodontics. Int. Endod. J. 18, 109–118. 10.1111/j.1365-2591.1985.tb00427.x 2860072

[B4] Andreasen J. O. Flores M. T. (2007). Injuries to developing teeth, in Textbook and Color Atlas of Traumatic Injuries to the Teeth, ed Andreasen J. O. Andreasen F. M. Andersson L. (Oxford: Blackwell Munksgaard), 542–576.

[B5] Andreasen J. O. Sundström B. Ravn J. J. (1971). The effect of traumatic injuries to primary teeth on their permanent successors. I. A clinical and histologic study of 117 injured permanent teeth. Scand. J. Dent. Res. 79, 219–283. 5286029 10.1111/j.1600-0722.1971.tb02013.x

[B6] Ansari G. Reid J. S. (1997). Dentinal dysplasia type I: review of the literature and report of a family. J. Dent. Child. 64, 429–434. 9466016

[B7] Bae C. H. Kim T. H. Ko S. O. Lee J. C. Yang X. Cho E. S. (2015). Wntless regulates dentin apposition and root elongation in the mandibular molar. J. Dent. Res. 94, 439–445. 10.1177/0022034514567198 25595365 PMC4814015

[B8] Baer P. N. Brown N. C. Hamner J. E. (1964). Hypophosphatasia: report of two cases with dental findings. Periodontics 2, 209–215.

[B9] Bafna Y. Kambalimath H. V. Khandelwal V. Nayak P. (2013). Taurodontism in deciduous molars. BMJ Case Rep. 2013:bcr2013010079. 10.1136/bcr-2013-010079 23737594 PMC3703011

[B10] Barbería E. Hernandez C. Miralles V. Maroto M. (2008). Paediatric patients receiving oncology therapy: review of the literature and oral management guidelines. Eur. J. Paediatr. Dent. 9, 188–194. 19072007

[B11] Bosshardt D. D. Stadlinger B. Terheyden H. (2015). Cell-to-cell communication - periodontal regeneration. Clin. Oral Implants Res. 26, 229–239. 10.1111/clr.12543 25639287

[B12] Carlos R. Contreras-Vidaurre E. de Almeida O. P. Silva K. R. Abrahão P. G. Miranda A. M. M. A.. (2008). Regional odontodysplasia: morphological, ultrastructural, and immunohistochemical features of the affected teeth, connective tissue, and odontogenic remnants. J. Dent. Child. 75, 144–150. 18647509

[B13] Chapple I. L. C. (1993). Hypophosphatasia: dental aspects and mode of inheritance. J. Clin. Periodontol. 20, 615–622. 10.1111/j.1600-051X.1993.tb00705.x 8227447

[B14] Crawford P. J. Aldred M. J. (1989). Regional odontodysplasia: a bibliography. J. Oral Pathol. Med. 18, 251–263. 10.1111/j.1600-0714.1989.tb00394.x 2549236

[B15] Da Rós Gonçalves L. Oliveira C. A. G. R. Holanda R. Silva-Boghossian C. M. Vieira Colombo A. P. Maia L. C.. (2008). Periodontal status of patients with dentin dysplasia type I: report of three cases within a family. J. Periodontol. 79, 1304–1311. 10.1902/jop.2008.070426 18597615

[B16] Diekwisch T. G. H. (2001). Developmental biology of cementum. Int. J. Dev. Biol. 45, 695–706. 11669371

[B17] Dineshshankar J. Sivakumar M. Balasubramanium A. M. Kesavan G. Karthikeyan M. Prasad V. S. (2014). Taurodontism. J. Pharm. Bioallied. Sci. 6, S13–S15. 10.4103/0975-7406.137252 25210354 PMC4157250

[B18] Dong J. Amor D. Aldred M. J. Gu T. Escamilla M. MacDougall M. (2005). DLX3 mutation associated with autosomal dominant amelogenesis imperfecta with taurodontism. Am. J. Med. Genet. 133A, 138–141. 10.1002/ajmg.a.30521 15666299

[B19] Duverger O. Zah A. Isaac J. Sun H.-W. Bartels A. K. Lian J. B.. (2012). Neural crest deletion of Dlx3 leads to major dentin defects through down-regulation of Dspp. J. Biol. Chem. 287, 12230–12240. 10.1074/jbc.M111.326900 22351765 PMC3320974

[B20] El-Labban N. G. Lee K. W. Rule D. (1991). Permanent teeth in hypophosphatasia: light and electron microscopic study. J. Oral Pathol. Med. 20, 352–360. 10.1111/j.1600-0714.1991.tb00944.x 1895252

[B21] Fearne J. Williams D. M. Brook A. H. (1986). Regional odontodysplasia: a clinical and histological evaluation. J. Int. Assoc. Dent. Child. 17, 21–25. 2822816

[B22] Fujiwara N. Akimoto T. Otsu K. Kagiya T. Ishizeki K. Harada H. (2009). Reduction of Egf signaling decides transition from crown to root in the development of mouse molars. J. Exp. Zool. B Mol. Dev. Evol. 312B, 486–494. 10.1002/jez.b.21268 19090534

[B23] Gardner D. G. Sapp J. P. (1973). Regional odontodysplasia. Oral Surg. Oral Med. Oral Pathol. 35, 351–365. 10.1016/0030-4220(73)90073-X 4510607

[B24] Gardner D. G. Sapp J. P. (1977). Ultrastructural, electron-probe, and microhardness studies of the controversial amorphous areas in the dentin of regional odontodysplasia. Oral Surg. Oral Med. Oral Pathol. 44, 549–559. 10.1016/0030-4220(77)90298-5 198720

[B25] Gerlach R. F. Jorge J. de Almeida O. P. Della Coletta R. Zaia A. A. (1998). Regional odontodysplasia. Report of two cases. Oral Surg. Oral Med. Oral Pathol. 85, 308–313. 10.1016/S1079-2104(98)90014-2 9540089

[B26] Gibbard P. D. Lee K. W. Winter G. B. (1973). Odontodysplasia. Br. Dent. J. 135, 525–532. 10.1038/sj.bdj.4803111 4358870

[B27] Hamdan M. A. Sawair F. A. Rajab L. D. Hamdan A. M. Al-Omari I. K. H. (2004). Regional odontodysplasia: a review of the literature and report of a case. Int. J. Paediatr. Dent. 14, 363–370. 10.1111/j.1365-263X.2004.00548.x 15331002

[B28] Han X. L. Liu M. Voisey A. Ren Y. S. Kurimoto P. Gao T.. (2011). Post-natal effect of overexpressed DKK1 on mandibular molar formation. J. Dent. Res. 90, 1312–1317. 10.1177/0022034511421926 21917600 PMC3188462

[B29] Haridoss S. K. Swaminathan K. Rajendran V. Rajendran B. (2014). Single-rooted primary first mandibular molar. BMJ Case Rep. 2014:bcr2014206347. 10.1136/bcr-2014-206347 25150245 PMC4154040

[B30] Haskova J. E. Gill D. S. Figueiredo J. A. P. Tredwin C. J. Naini F. B. (2009). Taurodontism - a review. Dent. Update 36, 235–243. 19518034 10.12968/denu.2009.36.4.235

[B31] Hattab F. N. (2014). Double talon cusps on supernumerary tooth fused to maxillary central incisor: review of literature and report of case. J. Clin. Exp. Dent. 6, e400–e407. 10.4317/jced.51428 25593664 PMC4282909

[B32] Hu J. C. C. Plaetke R. Mornet E. Zhang C. Sun X. Thomas H. F.. (2000). Characterization of a family with dominant hypophosphatasia. Eur. J. Oral Sci. 108, 189–194. 10.1034/j.1600-0722.2000.108003189.x 10872988

[B33] Huang X. Bringas P. Jr. Slavkin H. C. Chai Y. (2009). Fate of HERS during tooth root development. Dev. Biol. 334, 22–30. 10.1016/j.ydbio.2009.06.034 19576204 PMC2744848

[B34] Huang X. F. Chai Y. (2012). Molecular regulatory mechanism of tooth root development. Int. J. Oral Sci. 4, 177–181. 10.1038/ijos.2012.61 23222990 PMC3633063

[B35] Jafarzadeh H. Abbott P. V. (2007). Dilaceration: review of an endodontic challenge. J. Endod. 33, 1025–1030. 10.1016/j.joen.2007.04.013 17931926

[B36] Jaffe N. Toth B. B. Hoar R. E. Ried H. L. Sullivan M. P. McNeese M. D. (1984). Dental and maxillofacial abnormalities in long-term survivors of childhood cancer: effects of treatment with chemotherapy and radiation to the head and neck. Pediatrics 73, 816–823. 6728583

[B37] Kalk W. W. I. Batenburg R. H. K. Vissink A. (1998). Dentin dysplasia type I. Five cases within one family. Oral Surg. Oral Med. Oral Pathol. 86, 175–178. 10.1016/S1079-2104(98)90121-4 9720092

[B38] Kerebel L.-M. Kerebel B. (1983). Soft-tissue calcifications of the dental follicle in regional odontodysplasia: a structural and ultrastructural study. Oral Surg. Oral Med. Oral Pathol. 56, 396–404. 10.1016/0030-4220(83)90350-X 6314222

[B39] Kim J.-W. Simmer J. P. (2007). Hereditary dentin defects. J. Dent. Res. 86, 392–399. 10.1177/154405910708600502 17452557

[B40] Kim T. H. Bae C. H. Lee J. C. Kim J. E. Yang X. de Crombrugghe B.. (2015). Osterix regulates tooth root formation in a site-specific manner. J. Dent. Res. 94, 430–438. 10.1177/0022034514565647 25568170 PMC4814021

[B41] Kottoor J. Velmurugan N. (2013). Revascularization for a necrotic immature permanent lateral incisor: a case report and literature review. Int. J. Paediatr. Dent. 23, 310–316. 10.1111/ipd.12000 22994878

[B42] Kupczik K. Hublin J.-J. (2010). Mandibular molar root morphology in Neanderthals and Late Pleistocene and recent *Homo sapiens*. J. Hum. Evol. 59, 525–541. 10.1016/j.jhevol.2010.05.009 20719359

[B43] Larson E. K. Cahill D. R. Gorski J. P. Marks S. C. Jr. (1994). The effect of removing the true dental follicle on premolar eruption in the dog. Arch. Oral Biol. 39, 271–275. 10.1016/0003-9969(94)90116-3 8024490

[B44] Lee H.-S. Kim S.-H. Kim S.-O. Choi B.-J. Cho S.-W. Park W.. (2015). Microscopic analysis of molar-incisor malformation. Oral Surg. Oral Med. Oral Pathol. Oral Radiol. 119, 544–552. 10.1016/j.oooo.2014.10.013 25544405

[B45] Lee H.-S. Kim S.-H. Kim S.-O. Lee J.-H. Choi H.-J. Jung H.-S.. (2014). A new type of dental anomaly: molar-incisor malformation (MIM). Oral Surg. Oral Med. Oral Pathol. Oral Radiol. 118, 101–109. 10.1016/j.oooo.2014.03.014 24908600

[B46] Lepe X. Rothwell B. R. Banich S. Page R. C. (1997). Absence of adult dental anomalies in familial hypophosphatasia. J. Periodontal Res. 32, 375–380. 10.1111/j.1600-0765.1997.tb00547.x 9210091

[B47] Lind V. (1972). Short root anomaly. Scand. J. Dent. Res. 80, 85–93. 10.1111/j.1600-0722.1972.tb00268.x 4505388

[B48] Özer L. Karasu H. Aras K. Tokman B. Ersoy E. (2004). Dentin dysplasia type I: report of atypical cases in the permanent and mixed dentitions. Oral Surg. Oral Med. Oral Pathol. Oral Radiol. Endod. 98, 85–90. 10.1016/j.tripleo.2004.01.005 15243476

[B49] Luan X. Ito Y. Diekwisch T. G. H. (2006). Evolution and development of Hertwig's epithelial root sheath. Dev. Dyn. 235, 1167–1180. 10.1002/dvdy.20674 16450392 PMC2734338

[B50] Lundgren T. Westphal O. Bolme P. Modéer T. Norén J. G. (1991). Retrospective study of children with hypophosphatasia with reference to dental changes. Scand. J. Dent. Res. 99, 357–364. 10.1111/j.1600-0722.1991.tb01041.x 1754836

[B51] MacDougall M. Dong J. Acevedo A. C. (2006). Molecular basis of human dentin diseases. Am. J. Med. Genet. 140A, 2536–2546. 10.1002/ajmg.a.31359 16955410

[B52] Marks S. C. Jr. Cahill D. R. (1984). Experimental study in the dog of the non-active role of the tooth in the eruptive process. Arch. Oral Biol. 29, 311–322. 10.1016/0003-9969(84)90105-5 6586126

[B53] McKee M. D. Hoac B. Addison W. N. Barros N. M. T. Millán J. L. Chaussain C. (2013). Extracellular matrix mineralization in periodontal tissues: noncollagenous matrix proteins, enzymes, and relationship to hypophosphatasia and X-linked hypophosphatemia. Periodontol. 2000 63, 102–122. 10.1111/prd.12029 23931057 PMC3766584

[B54] McKee M. D. Nakano Y. Masica D. L. Gray J. J. Lemire I. Heft R.. (2011). Enzyme replacement therapy prevents dental defects in a model of hypophosphatasia. J. Dent. Res. 90, 470–476. 10.1177/0022034510393517 21212313 PMC3144124

[B55] Minicucci E. M. Lopes L. F. Crocci A. J. (2003). Dental abnormalities in children after chemotherapy treatment for acute lymphoid leukemia. Leuk. Res. 27, 45–50. 10.1016/S0145-2126(02)00080-2 12479851

[B56] Nakatomi M. Morita I. Eto K. Ota M. S. (2006). Sonic hedgehog signaling is important in tooth root development. J. Dent. Res. 85, 427–431. 10.1177/154405910608500506 16632755

[B57] Neumann F. Würfel F. Mundt T. (1999). Dentin dysplasia type I. A case report. Ann. Anat., 181, 138–140. 10.1016/S0940-9602(99)80120-4 10081578

[B58] O Carroll M. K. Duncan W. K. (1994). Dentin dysplasia type I. Radiologic and genetic perspectives in a six-generation family. Oral Surg. Oral Med. Oral Pathol. 78, 375–381. 7970601 10.1016/0030-4220(94)90071-x

[B59] O Carroll M. K. Duncan W. K. Perkins T. M. (1991). Dentin dysplasia: review of the literature and a proposed subclassification based on radiographic findings. Oral Surg. Oral Med. Oral Pathol. 72, 119–125. 10.1016/0030-4220(91)90202-N 1891231

[B60] Olsson A. Matsson L. Blomquist H. K. Larsson Å. Sjödin B. (1996). Hypophosphatasia affecting the permanent dentition. J. Oral Pathol. Med. 25, 343–347. 10.1111/j.1600-0714.1996.tb00274.x 8887081

[B61] Palit M. C. Hegde K. S. Bhat S. S. Sargod S. S. Mantha S. Chattopadhyay S. (2014). Tissue engineering in endodontics: root canal revascularization. J. Clin. Pediatr. Dent. 38, 291–297. 10.17796/jcpd.38.4.j5285857278615r1 25571677

[B62] Park J.-C. Herr Y. Kim H.-J. Gronostajski R. M. Cho M.-I. (2007). *Nfic* gene disruption inhibits differentiation of odontoblasts responsible for root formation and results in formation of short and abnormal roots in mice. J. Periodontol. 78, 1795–1802. 10.1902/jop.2007.060363 17760551

[B63] Pedersen L. B. Clausen N. Schrøder H. Schmidt M. Poulsen S. (2012). Microdontia and hypodontia of premolars and permanent molars in childhood cancer survivors after chemotherapy. Int. J. Paediatr. Dent. 22, 239–243. 10.1111/j.1365-263X.2011.01199.x 22092748

[B64] Price J. A. Bowden D. W. Wright J. T. Pettenati M. J. Hart T. C. (1998). Identification of a mutation in *DLX3* associated with tricho-dento-osseous (TDO) syndrome. Hum. Mol. Genet. 7, 563–569. 10.1093/hmg/7.3.563 9467018

[B65] Price J. A. Wright J. T. Walker S. J. Crawford P. J. M. Aldred M. J. Hart T. C. (1999). Tricho-dento-osseous syndrome and amelogenesis imperfecta with taurodontism are genetically distinct conditions. Clin. Genet. 56, 35–40. 10.1034/j.1399-0004.1999.550105.x 10466415

[B66] Ranta H. Lukinmaa P.-L. Waltimo J. (1993). Heritable dentin defects: nosology, pathology, and treatment. Am. J. Med. Genet. 45, 193–200. 10.1002/ajmg.1320450209 8456802

[B67] Rocha C. T. Nelson-Filho P. da Silva L. A. B. Assed S. de Queiroz A. M. (2011). Variation of dentin dysplasia type I: report of atypical findings in the permanent dentition. Braz. Dent. J. 22, 74–78. 21519653 10.1590/s0103-64402011000100013

[B68] Romito L. M. (2004). Concrescence: report of a rare case. Oral Surg. Oral Med. Oral Pathol. Oral Radiol. Endod. 97, 325–327. 10.1016/j.tripleo.2003.10.015 15024354

[B69] Sapp J. P. Gardner D. G. (1973). Regional odontodysplasia: an ultrastructural and histochemical study of the soft-tissue calcifications. Oral Surg. Oral Med. Oral Pathol. 36, 383–392. 10.1016/0030-4220(73)90216-8 4516466

[B70] Schroeder H. E. (1991). Oral Structural Biology. Stuttgart; New York, NY: Thieme Medical Publishers.

[B71] Schuurs A. H. B. van Loveren C. (2000). Double teeth: review of the literature. J. Dent. Child. 67, 313–325. 11068663

[B72] Shabahang S. (2013). Treatment options: apexogenesis and apexification. Pediatr. Dent. 35, 125–128. 10.1016/j.joen.2012.11.046 23635980

[B73] Shankly P. E. Mackie I. C. Sloan P. (1999). Dentinal dysplasia type I: report of a case. Int. J. Paediatr. Dent. 9, 37–42. 10.1046/j.1365-263x.1999.00106.x 10336715

[B74] Shashirekha G. Jena A. (2013). Prevalence and incidence of gemination and fusion in maxillary lateral incisors in Odisha population and related case report. J. Clin. Diagn. Res. 7, 2326–2329. 24298521 10.7860/JCDR/2013/5677.3516 PMC3843463

[B75] Shields E. D. Bixler D. el-Kafrawy A. M. (1973). A proposed classification for heritable human dentine defects with a description of a new entitiy. Arch. Oral Biol. 18, 543–553. 10.1016/0003-9969(73)90075-7 4516067

[B76] Sonis A. L. Tarbell N. Valachovic R. W. Gelber R. Schwenn M. Sallan S. (1990). Dentofacial development in long-term survivors of acute lymphoblastic leukemia. A comparison of three treatment modalities. Cancer 66, 2645–2652. 2249205 10.1002/1097-0142(19901215)66:12<2645::aid-cncr2820661230>3.0.co;2-s

[B77] Spini T. H. Sargenti-Neto S. Cardoso S. V. Souza K. C. N. de Souza S. O. M. de Faria P. R.. (2007). Progressive dental development in regional odontodysplasia. Oral Surg. Oral Med. Oral Pathol. Oral Radiol. Endod. 104, e40–e45. 10.1016/j.tripleo.2007.02.027 17613259

[B78] Steele-Perkins G. Butz K. G. Lyons G. E. Zeichner-David M. Kim H.-J. Cho M.-I.. (2003). Essential role for NFI-C/CTF transcription-replication factor in tooth root development. Mol. Cell. Biol. 23, 1075–1084. 10.1128/MCB.23.3.1075-1084.2003 12529411 PMC140704

[B79] Tananuvat N. Charoenkwan P. Ohazama A. Ketuda Cairns J. R. Kaewgahya M. Kantaputra P. N. (2014). Root dentin anomaly and a PLG mutation. Eur. J. Med. Genet. 57, 630–635. 10.1016/j.ejmg.2014.09.006 25281489

[B80] Tervonen S. A. Stratmann U. Mokrys K. Reichart P. A. (2004). Regional odontodysplasia: a review of the literature and report of four cases. Clin. Oral Investig. 8, 45–51. 10.1007/s00784-003-0245-0 14714196

[B81] Toomarian L. Mashhadiabbas F. Mirkarimi M. Mehrdad L. (2010). Dentin dysplasia type I: a case report and review of the literature. J. Med. Case. Rep. 4, 1. 10.1186/1752-1947-4-1 20205797 PMC2823758

[B82] Topouzelis N. Tsaousoglou P. Pisoka V. Zouloumis L. (2010). Dilaceration of maxillary central incisor: a literature review. Dent. Traumatol. 26, 335–341. 10.1111/j.1600-9657.2010.00915.x 20831640

[B83] Tronstad L. (1988). Root resorption - etiology, terminology and clinical manifestations. Endod. Dent. Traumatol. 4, 241–252. 10.1111/j.1600-9657.1988.tb00642.x 3078294

[B84] Tummers M. Thesleff I. (2003). Root or crown: a developmental choice orchestrated by the differential regulation of the epithelial stem cell niche in the tooth of two rodent species. Development 130, 1049–1057. 10.1242/dev.00332 12571097

[B85] Valladares Neto J. Rino Neto J. de Paiva J. B. (2013). Orthodontic movement of teeth with short root anomaly: should it be avoided, faced or ignored? Dental Press, J. Orthod. 18, 72–85. 10.1590/S2176-94512013000600012 24351153

[B86] Van den Bos T. Handoko G. Niehof A. Ryan L. M. Coburn S. P. Whyte M. P.. (2005). Cementum and dentin in hypophosphatasia. J. Dent. Res. 84, 1021–1025. 10.1177/154405910508401110 16246934

[B87] Vieira A. R. Modesto A. Cabral M. G. (1998). Dentinal dysplasia type I: report of an atypical case in the primary dentition. J. Dent. Child. 65, 141–144. 9617458

[B88] Wesley R. K. Wysocki G. P. Mintz S. M. Jackson J. (1976). Dentin dysplasia type I. Oral Surg. Oral Med. Oral Pathol. 41, 516–524. 10.1016/0030-4220(76)90279-6 1063351

[B89] Wigler R. Kaufman A. Y. Lin S. Steinbock N. Hazan-Molina H. Torneck C. D. (2013). Revascularization: a treatment for permanent teeth with necrotic pulp and incomplete root development. J. Endod. 39, 319–326. 10.1016/j.joen.2012.11.014 23402501

[B90] Witt C. V. Hirt T. Rutz G. Luder H. U. (2014). Root malformation associated with a cervical mineralized diaphragm - a distinct form of tooth abnormality? Oral Surg. Oral Med. Oral Pathol. Oral Radiol. 117, e311–e319. 10.1016/j.oooo.2013.06.030 24018126

[B91] Wright J. T. Hong S. P. Simmons D. Daly B. Uebelhart D. Luder H. U. (2008). *DLX3* c.561_562delCT mutation causes attenuated phenotype of tricho-dento-osseous syndrome. Am. J. Med. Genet. 146A, 343–349. 10.1002/ajmg.a.32132 18203197

[B92] Wright J. T. Kula K. Hall K. Simmons J. H. Hart T. C. (1997). Analysis of the tricho-dento-osseous syndrome genotype and phenotype. Am. J. Med. Genet. 72, 197–204. 9382143 10.1002/(sici)1096-8628(19971017)72:2<197::aid-ajmg14>3.3.co;2-0

[B93] Xiong J. Gronthos S. Bartold P. M. (2013). Role of the epithelial cell rests of Malassez in the development, maintenance and regeneration of periodontal ligament tissues. Periodontol. 2000 63, 217–233. 10.1111/prd.12023 23931062

[B94] Xue Y. Wang W. Mao T. Duan X. (2012). Report of two Chinese patients suffering from *CLCN7*-related osteopetrosis and root dysplasia. J. Craniomaxillofac. Surg. 40, 416–420. 10.1016/j.jcms.2011.07.014 21962762

[B95] Yang J. Wang S.-K. Choi M. Reid B. M. Hu Y. Lee Y.-L.. (2015). Taurodontism, variations in tooth number, and misshapened crowns in Wnt10a null mice and human kindreds. Mol. Genet. Genomic Med. 3, 40–58. 10.1002/mgg3.111 25629078 PMC4299714

[B96] Yokohama-Tamaki T. Ohshima H. Fujiwara N. Takada Y. Ichimori Y. Wakisaka S.. (2006). Cessation of Fgf10 signaling, resulting in a defective dental epithelial stem cell compartment, leads to the transition from crown to root formation. Development 133, 1359–1366. 10.1242/dev.02307 16510502

[B97] Zarina R. S. R. Nik-Hussein N. N. (2005). Dental abnormalities of a long-term survivor of a childhood hematological malignancy: literature review and report of a case. J. Clin. Pediatr. Dent. 29, 167–174. 10.17796/jcpd.29.2.hq7307703428nt3v 15719924

[B98] Zweifler L. E. Patel M. K. Nociti F. H. Jr. Wimer H. F. Millán J. L. Somerman M. J.. (2015). Counter-regulatory phosphatases TNAP and NPP1 temporally regulate tooth root cementogenesis. Int. J. Oral Sci. 7, 27–41. 10.1038/ijos.2014.62 25504209 PMC4817535

